# The Separation of Emulsified Water/Oil Mixtures through Adsorption on Plasma-Treated Polyethylene Powder

**DOI:** 10.3390/ma14051086

**Published:** 2021-02-26

**Authors:** Asma Abdulkareem, Anton Popelka, Patrik Sobolčiak, Aisha Tanvir, Mabrouk Ouederni, Mariam A. AlMaadeed, Peter Kasak, Samer Adham, Igor Krupa

**Affiliations:** 1 Center for Advanced Materials, Qatar University, P.O. Box 2713 Doha, Qatar; asma.alkareem@qu.edu.qa (A.A.); anton.popelka@qu.edu.qa (A.P.); patrik@qu.edu.qa (P.S.); aisha.tanvir1991@gmail.com (A.T.); m.alali@qu.edu.qa (M.A.A.); peter.kasak@qu.edu.qa (P.K.); 2 QAPCO R&D–Qatar Petrochemical Company, P.O. Box 756 Doha, Qatar; MOuederni@qapco.com.qa; 3 GWSC-ConocoPhillips, Qatar Science & Technology Park, Tech 2 Building, No.109, P.O. Box 24750 Doha, Qatar; Samer.Adham@conocophillips.com

**Keywords:** oil/water emulsions, water treatment, adsorption, polyethylene, plasma treatment

## Abstract

This paper addresses the preparation and characterization of efficient adsorbents for tertiary treatment (oil content below 100 ppm) of oil/water emulsions. Powdered low-density polyethylene (LDPE) was modified by radio-frequency plasma discharge and then used as a medium for the treatment of emulsified diesel oil/water mixtures in the concentration range from 75 ppm to 200 ppm. Plasma treatment significantly increased the wettability of the LDPE powder, which resulted in enhanced sorption capability of the oil component from emulsions in comparison to untreated powder. Emulsions formed from distilled water and commercial diesel oil (DO) with concentrations below 200 ppm were used as a model of oily polluted water. The emulsions were prepared using ultrasonication without surfactant. The droplet size was directly proportional to sonication time and ranged from 135 nm to 185 nm. A sonication time of 20 min was found to be sufficient to prepare stable emulsions with an average droplet size of approximately 150 nm. The sorption tests were realized in a batch system. The effect of contact time and initial oil concentrations were studied under standard atmospheric conditions at a stirring speed of 340 rpm with an adsorbent particle size of 500 microns. The efficiency of the plasma-treated LDPE powder in oil removal was found to be dependent on the initial oil concentration. It decreased from 96.7% to 79.5% as the initial oil concentration increased from 75 ppm to 200 ppm. The amount of adsorbed oil increased with increasing contact time. The fastest adsorption was observed during the first 30 min of treatment. The adsorption kinetics for emulsified oils onto sorbent followed a pseudo-second-order kinetic model.

## 1. Introduction

Wastewaters from various sources of the petroleum industry (gas, crude oil, shale gas extraction, and oil refineries) represent the largest amounts of oily polluted water [1]. For example, the global production of processing wastewater was 202 billion barrels in 2014, and it is predicted to reach roughly 340 billion barrels in 2020 [2]. Wastewaters from the petroleum industry consist of both low molecular unsaturated and saturated hydrocarbons, and aromatic compounds such as benzene, toluene, xylene, and polyaromatic hydrocarbons [3]. These compounds have a mostly nonpolar character. On the other hand, wastewaters from the food industry and agriculture contain more polar natural oils and waxes [4]. The type of a treatment of oily polluted wastewaters depends on the oil concentration and can be categorized as primary, secondary, and tertiary treatment. Tertiary treatment is the purification of wastewater having approximately 70–100 ppm oil components, and the required breakthrough concentration is around 5–10 ppm, dependently on national regulations. Oil removal by sorption through adsorption (often accomplished by coalescence) using batch systems and percolating column configurations represents a fast and economically advantageous route for the tertiary treatment of oily polluted waters [1,5,6]. Because of fine powders, which are useful in batch systems due to significant pressure drop and are not suitable for deep-bed filtration, the adsorbents applicable in deep-bed filtration have mostly granular form [7,8].

Commonly used sorbents can be prepared from suitable natural products (walnut shell, pecan, chitosan-rich structures) or synthetic polymers which have controlled geometry and surface characteristics. Polymeric materials seem to be the most promising sorbents in general because of their low cost, selectivity, and variability of their morphology. The surface of these materials can be additionally modified through various chemical and physical methods [9].

Plasma treatment represents the most powerful tool for the surface modification of polymeric surfaces and is frequently used in many polymer-oriented industry applications [10]. Plasma treatment represents a dry, clean, eco-friendly technique for the surface modification of various materials (polymer, metal, wood, glass, etc.) [11]. The interactions of plasma-created species with a polymer surface can lead to different processes depending on the conditions used, such as the gas/gas mixture (Ar, N_2_, O_2_, CO_2_, NH_3_) and processing parameters (pressure, nominal power, treatment time, gas flow rate) [12]. The introduction of chemical functionalities can occur as a result of plasma oxidation, amination or nitration, while using gases without susceptibility for the formation of polymerizable intermediates after excitation. Moreover, free radical formation on a polymer surface can lead to surface activation, etching, ablation, or crosslinking processes [13]. Plasma treatment is thus responsible for changes in chemical composition accompanied by changes in topography/roughness. Moreover, it allows further interactions with other low- and high-molecular-weight species, and, therefore, enables modification of surfaces by various compounds (grafting) with desirable functionalities. Since all these changes are realized only in the top surface layer, the original physical properties are unchanged. All these modifications are realized on the final product, which is a very suitable technological route [14,15].

This paper is focused on the separation of oily components from emulsified water/diesel oil (DO) mixtures with oil contents of up to 200 ppm by plasma-treated low-density polyethylene (LDPE) powder in batch configurations. Plasma treatment was performed to improve the wettability of the LDPE sorbent by the emulsion, and, thus, to enhance its adsorption capacity.

## 2. Materials and Methods

### 2.1. Materials

Low-density polyethylene (LDPE) Lotrene FB3003 (MFI = 0.3 g/10 min, 190 °C, 2.16 kg; specific density = 0.92 g/cm^3^) in granular form supplied by Qatar Petrochemical Company (QAPCO, Doha, Qatar) was used as a raw material. LDPE granules were ground into powder form and sieved to obtain fractions of different sizes. A fraction with an average diameter (lateral dimension) of 0.5 mm was used for all experiments.

Ethanol (Sigma Aldrich, Darmstadt, Germany), ultrapure water (Purification System Direct Q3, Molsheim, France), and commercial diesel oil (DO) (petrol distribution company Woqod, Qatar) were used. DO is mostly composed of alkanes (C10–C32), as determined by gas chromatography.

### 2.2. Preparation of Emulsions

A mixture of 500 mL distilled water and 200 ppm DO was sonicated for 15 min at 40% amplitude using an ultrasonic sonicator (HIELSCHER UP400S, Berlin, Germany) device with a 22 mm titanium probe used as the homogenizer. Then, the stock solution was diluted with deionized water to prepare the required concentrations.

### 2.3. Plasma Treatment

Low-temperature plasma treatment of LDPE powder was performed using a Venus75-HF radio frequency (RF) plasma system (Plasma Etch Inc., Carson, CA, USA) in an air under vacuum. During plasma treatment, plasma-created reactive species were generated by means of an RF power supply operating at a typical frequency of 13.56 MHz. The chamber of the plasma system was evacuated to a pressure of approximately 0.2 Torr using a rotary vacuum pump prior to plasma application. Optimization of the treatment process was performed at various treatment times (10–180 s) to obtain the optimal wettability of the plasma-treated LDPE film. The optimized plasma treatment was then applied to the treat the LDPE samples in powder form, which were placed in closed Petri dishes wrapped by paraffin film during the plasma treatment in air, while the samples were turned over several times to ensure homogenous treatment for each side.

### 2.4. Surface Wettability Analysis

An OCA35 optical system (DataPhysics, Filderstadt, Germany) equipped with a CCD camera was employed to measure the wettability of the flat LDPE surfaces (films) after plasma treatment using static contact angle measurements via the sessile drop technique. Liquids with different surface tensions were tested to characterize the wettability of PE by an assessment of the surface free energy (γ) and its dispersive (γ_d_) and polar (γ_p_) components by using the Owens-Wendt-Rabel-Kaelble regression model [16].

### 2.5. Surface Morphology/Topography Analysis

The surface morphology of the untreated and treated LDPE (powder) samples was characterized with a field emission scanning electron microscope (FEI-SEM, Nova Nano SEM 450, Hillsboro, OR, USA) facilitated by energy-dispersive X-ray spectroscopy (EDS) using secondary electron images at 3 kV and varying magnification. The specimens were sputter-coated with an approximately 2 nm layer of gold before taking SEM images to avoid the accumulation of electrons in the measured layer and to obtain SEM images with high resolution.

The surface topography of the LDPE powder samples was characterized by profilometry (The Optical Surface Metrology System Leica DCM8, Mannheim, Germany). This system allows measuring the 3D surface topography of larger surface areas with no limits to the roughness. It contains five objectives with different magnifications (5×, 10×, 20×, 50×, 100×), allowing for analysis of samples using different-size areas, and a highly sensitive detector (1.4 million pixel resolution) was used for obtaining confocal images. An EPI 100X-L objective (1360 × 1024 data points) was used to obtain the maximal detailed images from a surface area of 175.31 × 131.97 μm^2^.

### 2.6. Surface Area Measurements

A BET surface area analyzer (Micromeritics-TriStar, Norcross (Atlanta), GA, USA) was employed to measure the specific surface area and pore size of the chosen grinded fraction of LDPE. The Brunauer-Emmet-Teller (BET) multipoint approach was employed to assess the surface area and pore distribution through nitrogen gas. The sample specific surface area was extrapolated at low temperatures of 70 °C from the amount of nitrogen (extremely small molecule) adsorbed onto the LDPE sample layer.

### 2.7. Chemical Composition Investigation

FTIR was used to qualitatively evaluate changes in the chemical composition of LDPE untreated and plasma-treated surfaces. For this analysis, an FTIR spectrometer frontier (PerkinElmer, Waltham, MA, USA) fitted with a ZnSe crystal was utilized, while the penetration depth of the infrared light was 1.66 μm. In addition, the spectral resolution and number of scans were set to 4 and 8, respectively. Qualitative information was obtained for the absorption of chemical groups in the middle infrared region (4000–500 cm^−1^).

X-ray photoelectron spectroscopy (XPS) was used for deeper characterization of the chemical composition changes induced by plasma treatment of LDPE powder. XPS spectra were captured using an Axis ultra DLD system (Kratos Analytical, UK) containing an Al Ka X-ray source. The sampling depth was in the range of 1–10 nm, allowing one to analyze only the top layer affected by plasma treatment (a few tens of nm).

### 2.8. Sorption of Oil from Emulsions

In this study, the mass of the LDPE powder for all the experiments was arbitrarily chosen to be 3 g, and the volume of the tested emulsion was 40 mL. The mass of the powder was selected to respect the volume/mass ratio of the used vial. For example, 3 g of powder occupied approximately 2/3 of the total volume of the vial (45 mL).

### 2.9. Total Organic Carbon (TOC) Analysis

TOC analysis was realized using a Formacs TOC/TN analyzer (Skalar Analyzer, Breda, The Netherlands). The samples were injected into a high-temperature combustion furnace where organic carbon (OC) was converted to carbon dioxide at 850 °C by catalytic oxidation (Pt catalyst). The formed CO_2_ was then dispersed into the carrier gas, and the concentration was measured by using a nondispersive infrared detector (NDIR). The total inorganic carbon (TIC) was determined by injecting the sample into a reactor containing acid (H_3_PO_4_) converting TIC into carbon dioxide. The concentration of the related CO_2_ was then determined by NDIR. Finally, TOC was calculated by subtracting TIC from TC.

## 3. Results

### 3.1. Wettability of LDPE

**Figure 1:**
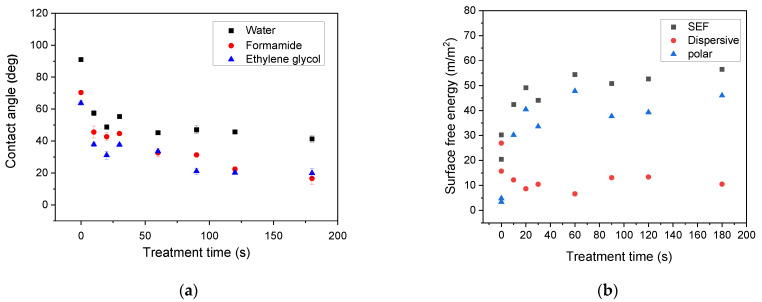
Dependence of (**a**) contact angle and (**b**) surface free energy (SFE) of selected liquids on plasma treated low-density polyethylene (LDPE) foil on treatment time.

Prior to plasma treatment of the LDPE powders, optimization of the processing conditions was realized through plasma treatment of LDPE films and wettability analysis. Plasma treatment time was in the range from 10 s to 180 s at a constant nominal power of 80 W. The contact angles and surface free energy for water, formamide, and ethylene glycol on the LDPE films are shown in Figure 1a,b. The contact angles of the untreated LDPE surface achieved relatively high values, indicating hydrophobic character (high wettability). Plasma treatment was responsible for the decrease in contact angles with the increase in treatment time as a result of the formation of polar functionalities on the LDPE surface. After reaching 60 s of plasma treatment time, only slight changes in contact angles were observed. Therefore, this treatment time was also applied for plasma treatment of LDPE powder.

**Figure 2:**
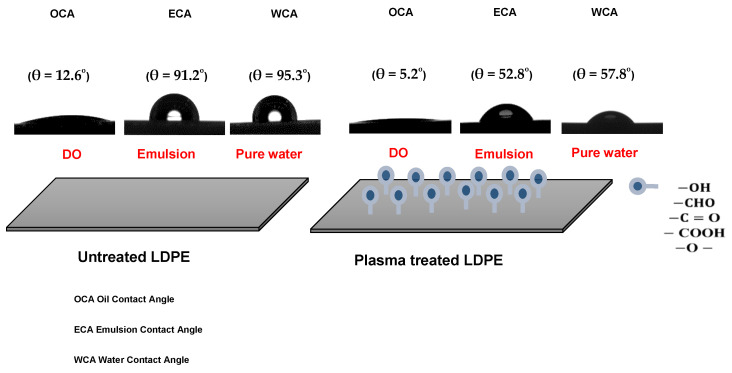
Illustration of the surface wettability of LDPE films.

**Figure 3:**
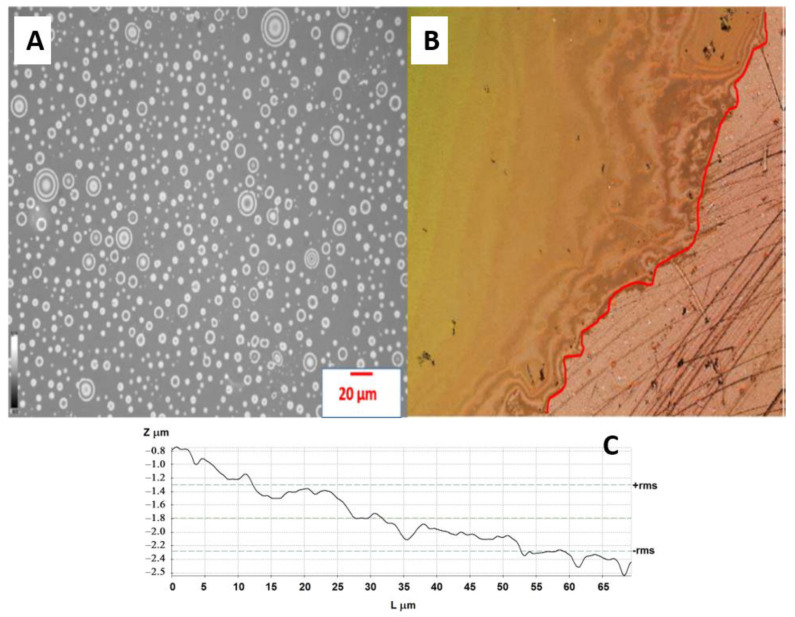
(**A**) The distribution of droplets of emulsion on the surface of treated PE foil; (**B**) Thin layer of DO deposited onto the surface of treated PE foil. The red line denotes the interface between the area covered by DO and the reference (non-immersed part of the foil); (**C**) Line profile for the surface of the treated PE foil.

The untreated and plasma-treated PE films were also analyzed in terms of their surface wettability by water, DO, and 100 ppm emulsion. The untreated LDPE surface showed hydrophobic and oleophilic characteristics; the water contact angle (WCA), oil contact angle (OCA) and emulsion contact angle (ECA) showed values of 95.3, 91.2, and 12.6°, respectively (Figure 2). The plasma treatment resulted in a significant improvement in wettability, while the WCA and ECA decreased to 57.8° and 52.8°, respectively. Moreover, OCA achieved 5.2° indicating very high oleophilicity. PE foil immersed into the DO emulsion shows no visible attachment of the oil droplets and DO droplets were freely moving (Figure 3A). On the other hand, PE film immersed in DO formed a continuously covered oil layer with an average thickness of 1.4 ± 0.1 μm, as determined by profilometry. These preliminary findings anticipate the applicability of plasma for improving oil/water separation.

### 3.2. Characterization of LDPE Powder

**Figure 4:**
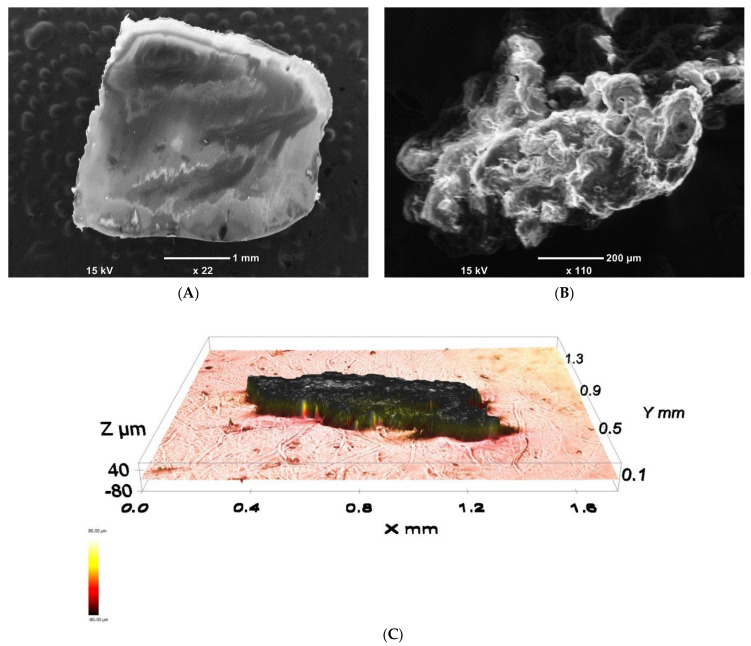
SEM micrograph of a common as-produced LDPE pellet (**A**) SEM micrograph; (**B**) and profilometry image (**C**) of LDPE powder prepared by grinding.

Unlike LDPE pellets, which always have smooth surfaces as a consequence of the route of preparation (extrusion) (Figure 4A), LDPE powders, depending on the route of the preparation (mostly grinding; partly precipitation from a solution), can have very porous structures, as demonstrated by SEM and profilometry analysis and shown in Figure 4B,C. The specific surface area of the powder was determined by BET analysis to be 44 ± 1 m^2^/g.

A comparison of a simple estimation of the specific surface area of smooth pellets and porous grinded powders demonstrates enhancement of the specific surface of the ground powders. The pellet is approximated by a perfectly smooth sphere with diameter D = 500 μm, which corresponds to the size of the powder used in this study. The specific surface area of perfectly smooth spheres (*S_a_*) can be calculated from Equation (1):

(1) $$s_{a}=\frac{6}{\rho D}$$

*D* is the diameter of the uniform spheres, and *ρ* is the bulk density of the material. In our case, for *D* = 500 μm and *ρ* = 0.92 g·cm^−3^, and, therefore *S_a_* = 0.013 m^2^/g, the experimentally determined value for the specific surface area of powder is 4.7 m^2^/g. This value is 362 times higher than the surface area of smooth spheres of the same size. This supports the statement that grinding neat pellets significantly enhances the surface porosity of materials, and, thus, enhances the surface area of powders.

**Figure 5:**
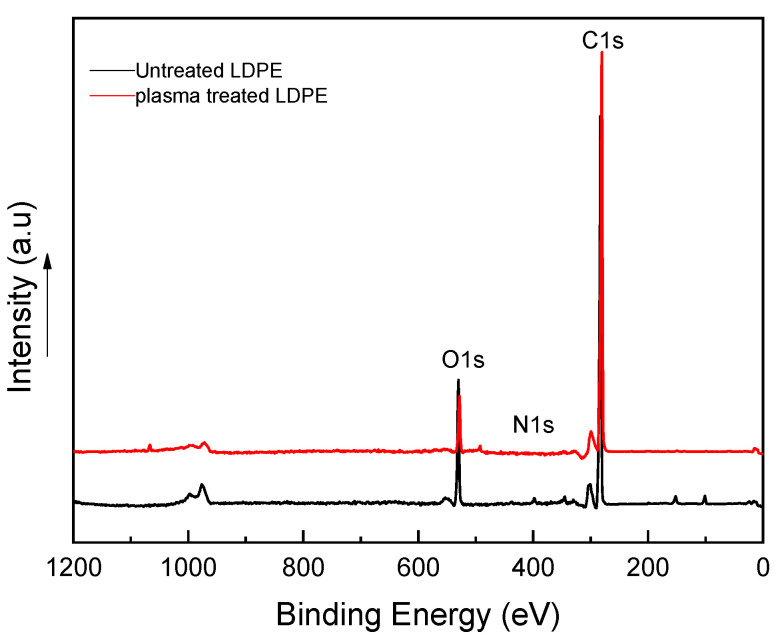
X-ray photoelectron spectroscopy (XPS) spectra for treated and untreated LDPE samples.

X-ray photoelectron spectroscopy (XPS) was employed to quantify the changes in the surface chemical composition of LDPE samples before and after plasma treatment in more detail. The XPS spectra measured for LDPE powder prior to and after treatment are shown in Figure 5. The quantification report for the atomic compositions of all samples is listed in Table 1. All samples were investigated for the C 1s, O 1s, and N 1s spectra. The highest proportion in the XPS spectrum of untreated LDPE samples was reflected by the C 1s peak at ~280 eV. As shown in Table 1, the spectrum of untreated LDPE powder revealed high carbon atomic concentrations (at.%) equal to 99.0 at.%. In addition, negligible traces of oxygen and nitrogen-containing functional groups present in the spectra of untreated LDPE samples are likely attributed to processing additives or residual air within the plasma chamber [17]. The atomic concentrations of the O 1s and N 1s peaks for untreated LDPE are equal to 0.5 at.% and 0.5 at.%, respectively. Plasma treatment led to an increase in the intensity of the O 1s and N 1s peaks at binding energies of ~528 eV and ~400 eV, respectively, as illustrated in Figure 5. The O 1s and N 1s atomic percentages increased by 9.8% and 0.8% in powder LDPE post-treatment, respectively. Nonetheless, this increase led to a reduction in the intensity of the C 1s peak to 90.2%. This effect is a result of the loss of some carbons throughout etching, radicalization, and substitution with groups containing oxygen [17]. Furthermore, as confirmed by Arpagaus et al. [18], the increase in peak intensity for powder LDPE is proof of an effective and homogeneous treatment, which is due to close contact between the powder surface and the plasma-created species [19].

### 3.3. Characterization of DO/Water Emulsions

**Figure 6:**
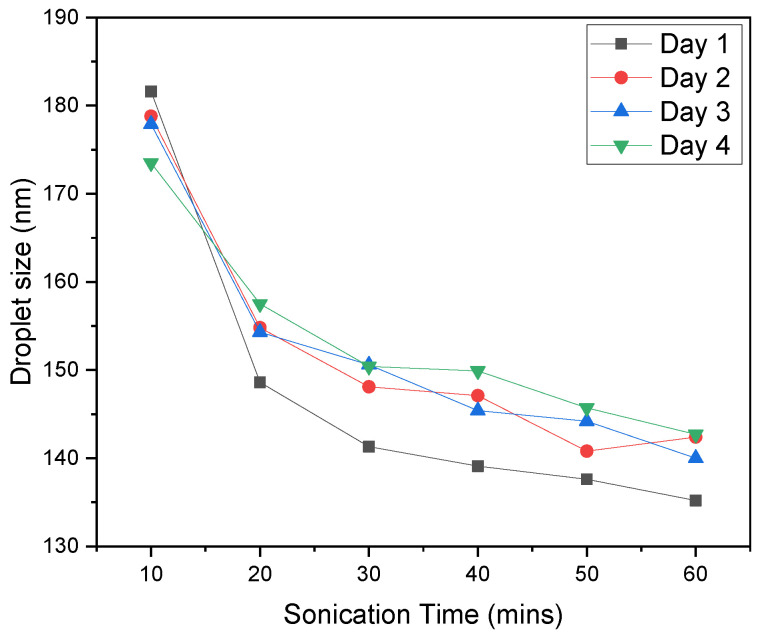
The dependence of the droplet size on the sonication time and the duration of emulsion storage (until four days).

Emulsions are thermodynamically unstable systems due to their natural tendency to minimize interfacial interactions between chemically heterogeneous components [20,21]. From a practical point of view (for instance, because of a delay between emulsion preparation, storage, and testing), it is important to know whether or not emulsions are stable over time. The evolution of droplet size over time is the key parameter for the stability of emulsion estimation since the instability is affected by changes in droplet size [22]. The stability of oil/water emulsions was inspected by Dynamic light scattering (DLS), directly determining the size of the droplets. The results are summarized in Figure 6. The droplet size remains fairly consistent over four days and is directly proportional to sonication time. A higher sonication time results in a smaller droplet size. To prepare a stable emulsion, twenty minutes of sonication was sufficient.

### 3.4. The Influence of Initial Oil Content

The initial oil portion in the emulsion can influence the kinetics of the adsorption, and, therefore, this parameter has to be taken into account [23]. The influence of the initial DO concentration on the adsorption process under arbitrary conditions (3.0 g of adsorbent, 24 h, stirring) was investigated by varying the initial concentration from 75 ppm to 200 ppm. The results are summarized in Table 2. It is evident that the quantity of oil adsorbed per unit weight of adsorbents q_e_ increases with an increase in the initial oil content. On the other hand, the oil removal efficiency decreased from 96.7% to 79.5% as the initial oil concentration of the DO emulsion increased from 75 ppm to 200 ppm. This phenomenon can be caused by the saturation of the available adsorption sites at higher oil concentrations, which means that equilibrium between adsorbed oil and oil in the emulsion is reached at higher concentrations of oil in the emulsion. Similar results have been achieved by Okie et al. 2011 [24] and Dirak et al. 2018 [25].

#### 3.4.1. Adsorption Isotherms

**Figure 7:**
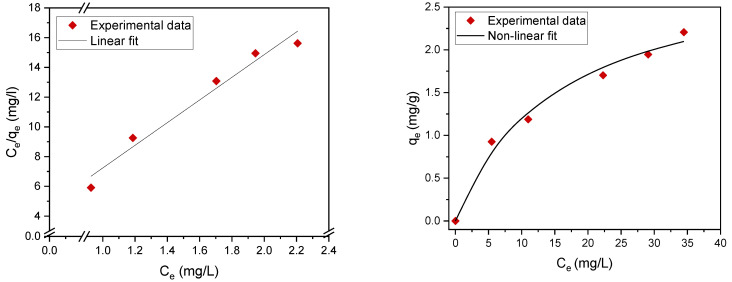
Langmuir adsorption isotherm nonlinear fit (**right**) and linear fit (**left**).

**Figure 8:**
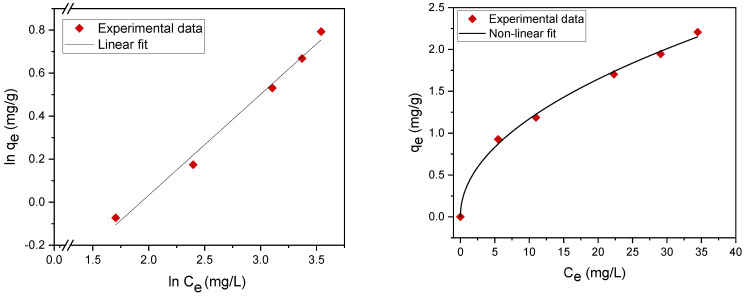
Freundlich adsorption isotherm nonlinear fit (**right**) and linear fit (**left**).

Adsorption isotherms contribute to the effective construction of adsorption systems, because they assess the maximum adsorption capacity that can be attained throughout the treatment process. The experimental results were evaluated using two of the most common isotherms, namely, the Langmuir [26,27,28,29] and Freundlich adsorption isotherms [30,31,32] (Table 3). The maximum adsorption capacity q_m_ (mg/g) and adsorption (equilibrium) constants were determined. The fitting data are summarized in Table 4 and Figure 7 and Figure 8. The results revealed that the experimental data can be well fitted by both the Langmuir and Freundlich isotherms with high correlation coefficients (R^2^), which indicates mixed monolayer and multilayer adsorption.

A useful parameter associated with the Langmuir isotherm is called the separation factor *R_L_* (Equation (2)):

(2) $$R_{L}=\frac{1}{1+K_{L}C_{0}}$$

where *R_L_* indicates the shape of the isotherms, *C*_0_ (mg/L) is the initial concentration, and *K_L_* (L/mg) is the Langmuir constant related to the energy of adsorption from emulsions [32]:

*R_L_* corresponds to the adsorption processes according to the following criteria [32]:

Case I. *R_L_* > 1: The adsorption is unfavorable (an increase in Gibbs free energy of adsorption).

Case II. 1 > *R_L_* > 0: The adsorption is favorable (a decrease in Gibbs free energy).

Case III. *R_L_* = 1 Characterization of a linear adsorption (unoccupied sites at the adsorbent are randomly occupied by adsorbate proportionally to their concentration, and only one reaction site is occupied by one species).

Case IV. *R_L_* = 0: The desorption process is irreversible.

The *R_L_* values are significantly lower than that found for all tested initial concentrations (C_0_), indicating highly favorable adsorption of oil droplets on the adsorbent.

Linear and nonlinear Freundlich isotherms are shown in Figure 8. The parameters (*K_F_*, 1/n) were determined from both linear and nonlinear fitting of the experimental data. The value of the exponent *n* > 1 indicates a favorable adsorption of oil on the plasma-treated PE powder [23].

*q_e_* is the amount of substance adsorbed at equilibrium per amount of adsorbent (mg/g), *C_e_* is the equilibrium concentration (mg/L), *q_m_* is the saturation adsorption capacity (mg/g), and *K_L_* is the Langmuir equilibrium adsorption constant (L/mg). 1/*n* is the heterogeneity factor, *n* characterizes the intensity of the adsorption process, the relative distribution of the energy and the heterogeneity of the adsorbent reactive sites, and *K_F_* (L/mg) is the Freundlich adsorption constant.

#### 3.4.2. Kinetics of Adsorption

**Figure 9:**
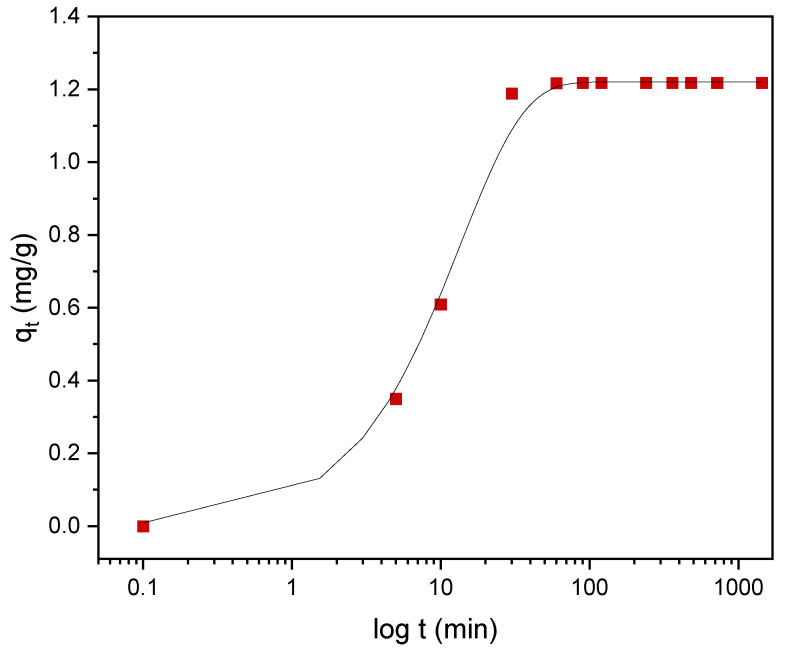
The dependence of the adsorption capacity (red squares) of DO on the contact time with plasma-treated LDPE. Black line represents the nonlinear, pseudo-first-order (PFO) kinetic model.

**Figure 10:**
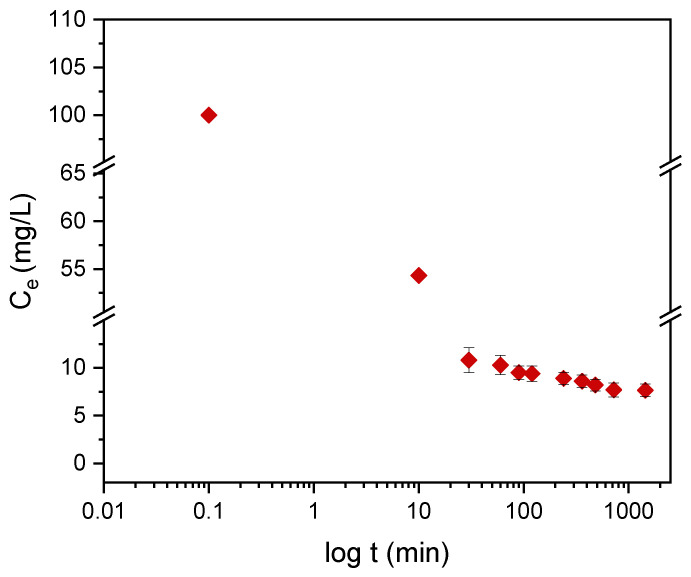
The dependence of the residual concentration of DO on the contact time with the plasma-treated LDPE powder.

**Figure 11:**
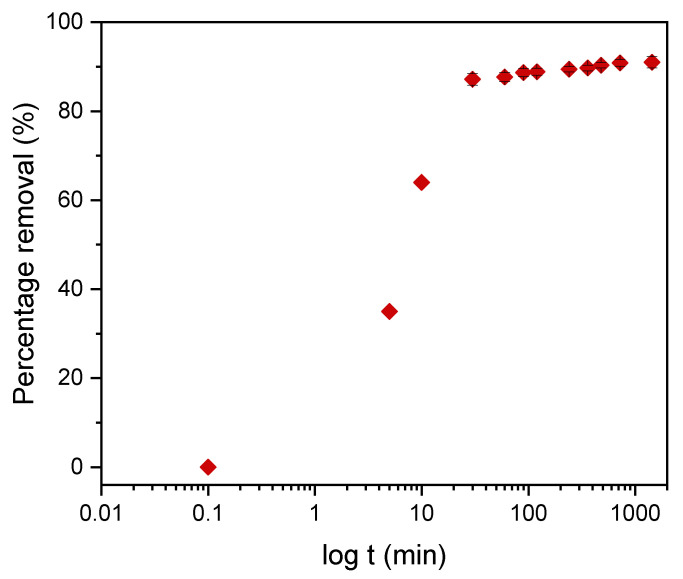
The dependence of the percentage of DO removal on the contact time with the plasma-treated LDPE power.

Kinetic models serve to estimate the duration of adsorption processes, and, thus, to estimate the time needed for the effective treatment of liquids. In batch systems, the solute concentration in the treated liquid gradually decreases with time until it reaches equilibrium with the adsorbed species. To determine the equilibrium time related to the maximum oil removal from emulsified oils [24], the amount of oil adsorbed onto the adsorbent was studied as a function of contact time in the range from 30 min to 1440 min using an initial oil concentration of 100 ppm and a treated adsorbent dose of 3.0 g. Equilibrium is achieved when the adsorption rate from the solution onto the surface of the sorbent corresponds to the rate of desorption from the sorbent to the solution [4]. As shown in Figure 9, the adsorption capacity (*q_t_*) increased over the whole duration of the sorption process; however, most of the oil was adsorbed within a short time (the first 15 min) of the treatment. Thereafter, the oil removal efficiency reaches equilibrium when the rate of adsorption and desorption are equilibrated. This does not necessarily mean that the whole surface area is occupied and that the surface of the adsorbent does not need to be saturated with the oil. A high removal efficiency of 91.0% was achieved. The amount of adsorbed oil is directly proportional to the other parameters: the residual concentration of oil in the emulsion (Figure 10) and the percentage of removal (Figure 11). The first parameter decreases over contact time, whereas the latter increases.

**Figure 12:**
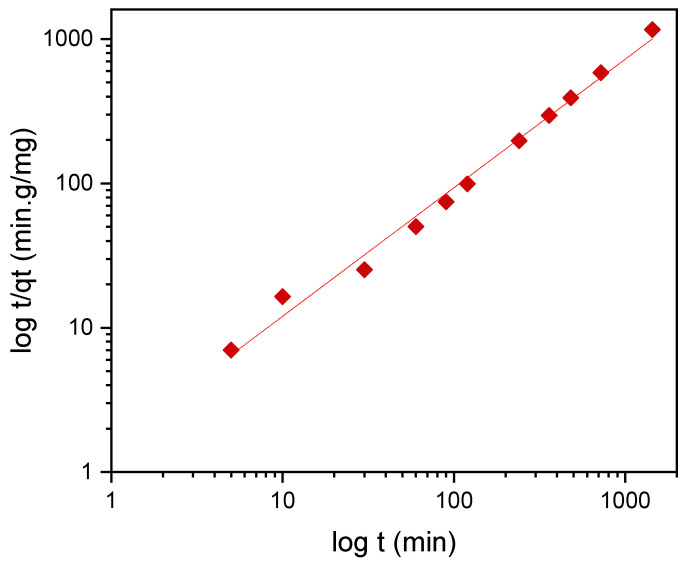
The linear pseudo-second-order kinetic model. Red squares—experimental t/q_t_ data.

To describe the adsorption kinetics, two widely used models, namely, the pseudo-first-order (PFO) and the pseudo-second-order (PSO) [33,34,35] kinetic models, were tested to fit the experimental data. The common linear and nonlinear forms of those equations are shown in Table 5. The nonlinear form of PFO was used to keep the parameter *q_e_* as the adjustable parameter (Figure 9). It was found that PFO does not provide good agreement with the experimental data. The parameters of the PSO model, *q_e_* and *k*_2_, were determined from linear fitting (Figure 12), and good agreement of the experimental data with the PSO model was demonstrated. All the parameters and constants determined from the PFO and PSO models are summarized in Table 6. The results confirm that the adsorption kinetics for emulsified oils onto powder LDPE follow the PSO kinetic model. As reported by various authors, the PSO model better describes the sorption kinetics at lower initial concentrations [34,35,36].

*q_t_* is the amount of adsorbed species per mass of adsorbent (mg/g), *k*_1_ (min^−1^) is the pseudo-first-order rate constant, *q_e_* is the amount of adsorbed species per mass of adsorbent in equilibrium (mg/g), and *t* is time (min). *k*_2_ is the pseudo-second-order rate constant. Unlike *k*_1_, which always has the dimension reciprocal to time, the constant *k*_2_ may have various dimensions (mg/g·min, g/g·min, mmol/g·min, etc.)

## 4. Conclusions

LDPE powder prepared by grinding LDPE pellets and treating with radio-frequency plasma discharge was used as a polymer-based adsorption medium for the treatment of emulsified water/oil mixtures. Plasma treatment significantly increased the wettability of the LDPE powder, which resulted in enhanced sorption efficiency. The sorption ability of untreated PE powder was also prechecked; however, due to a low oil removal efficiency, it was not investigated in detail, and, therefore, these results are not included in this paper. The findings can be summarized as follows:

1. Emulsions formed from distilled water and commercial DO with concentrations below 200 ppm were used as a model of oily polluted water. The emulsions were prepared without emulsifier, and emulsification was ensured by ultrasonication. The long-term stability of emulsions was demonstrated by determining the evolution of oil droplet size over time.
2. It was found that the plasma-treated LDPE surface exhibited a highly hydrophilic character due to the incorporation of new polar functionalities on the surface, related to the change of the atomic composition indicated by the XPS method.
3. The efficiency of the plasma-treated LDPE powder in oil removal was dependent on the initial oil concentration. It decreased from 96.7% to 79.5% as the initial oil concentration increased from 75 ppm to 200 ppm.
4. Freundlich isotherm better approximated the experimental points, which indicates mixed monolayer and multilayer adsorption.
5. The adsorbed amount of oil increased with increasing contact time. The fastest adsorption was observed during the first 30 min of treatment. The adsorption kinetics for emulsified oils onto the sorbent followed the pseudo-second order kinetic model.

## Figures and Tables

**Table 1:** XPS atomic composition summary for LDPE samples.

	Element	Atomic Conc. (at %)		
Samples		C 1s	O 1s	N 1s
Untreated powder		99.0	0.5	0.5
Treated powder		90.2	9.58	0.8

**Table 2:** Influence of initial oil concentration on the adsorption of emulsified diesel oil (DO) onto 3.0 g treated dosage at a contact time of 24 h.

Initial Oil Concentration C_0_ (mg/L)	Final Oil Concentration C_e_ (mg/L)	Oil Removed, C_0_-C_e_ (mg/L)	*q_e_ (mg/g)	Removal Efficiency (%)
75	5.5	70	0.93	96.7 ± 0.8
100	11.0	76.5	1.19	93.5 ± 0.9
100 **	25.5	89	0.77	66.4 ± 3.0
150	22.3	128	1.70	86.7 ± 1.5
175	29.1	146	1.95	82.7 ± 1.8
200	34.5	166	2.21	79.5 ± 0.5

*q_e_ oil adsorbed per unit weight of adsorbent. ** Removal efficiency of untreated LDPE powder determined under the same conditions.

**Table 3:** Nonlinear and linear forms of the Langmuir and Freundlich isotherms.

Equation Form	Langmuir Isotherm	Freundlich Isotherm
Nonlinear	$q_{e}\;=\;q_{m}\frac{K_{L}C_{e}}{1+K_{L}C_{e}\;}$	$q_{e}\;=\;K_{F}C_{e}^{\frac{1}{n}}$
Linear	$\frac{C_{e}}{q_{e}}=\frac{1}{K_{L}q_{m\;}}+\frac{C_{e}}{q_{m}}$	$\ln q_{e=}\ln K_{F\;}+\frac{1}{n}\;\ln C_{e}$

**Table 4:** Langmuir and Freundlich isotherm parameters.

Fitting	Langmuir Isotherm				Freundlich Isotherm		
	*q_m_* (mg/g)	*K_L_* (L/mg)	$\frac{1}{K_{L}q_{m}}$	R^2^	*K_F_* (L/mg)	n	R^2^
Nonlinear	3.04	0.06	5.48	0.989	0.377	2.03	0.996
Linear	3.03	0.066	4.97	0.968	0.404	2.13	0.987

**Table 5:** Nonlinear and linear forms of the PFO and pseudo-second-order (PSO) kinetic models.

Equation Form	PFO Model	PSO Model
Nonlinear	$q_{t\;}=q_{e}\left(1-e^{-k_{1}t}\;\right)$	$q_{t}=\frac{q_{e}^{2}k_{2\;}t}{1+q_{e}k_{2\;}t}$
Linear	$\ln\left(q_{e}-q_{t}\right)=lnq_{e}-k_{1}t$	$\frac{t}{q_{t}}=\frac{1}{q_{e}^{2}\;k_{2\;}}+\frac{t}{q_{e}}$

**Table 6:** Parameters of the PFO and PSO kinetic models.

PFO Model				PSO Model		
*q_e_* exp. (mg/g)	*q_e_* fit. (mg/g)s	*k*_1_ (s^−1^)	R^2^	*q_e_* fit. (mg/g)	*k*_2_ (g/min·mg)	R^2^
1.23	1.22	0.0741	0.841	1.23	0.7092	0.989

## Data Availability

The data presented in this study are available on request from the corresponding author.
